# Interferons in Colorectal Cancer Pathogenesis and Therapy

**DOI:** 10.54457/dr.202401005

**Published:** 2024-04-23

**Authors:** Lucy Petrova, Fred Bunz

**Affiliations:** 1 Department of Radiation Oncology and Molecular Radiation Sciences, Sidney Kimmel Comprehensive Cancer Center, Johns Hopkins University School of Medicine, Baltimore Maryland 21287, USA

**Keywords:** Interferons, Colorectal, Cancer, Therapy, P53

## Abstract

As key modulators of the immune response, interferons play critical roles following infection and during the pathogenesis of cancer. The idea that these cytokines might be developed as new therapies emerged soon after their discovery. While enthusiasm for this approach to cancer therapy has waxed and waned over the ensuing decades, recent advances in cancer immunotherapy and our improved understanding of the tumor immune environment have led to a resurgence of interest in this unique class of biologic drug. Here, we review how interferons influence the growth of colorectal cancers (CRCs) and highlight new insights into how interferons and drugs that modulate interferon expression might be most effectively deployed in the clinic.

## Introduction: Interferons Shape the Immune Responses

Interferons are multifunctional cytokines that serve as key mediators of innate and adaptive immunity. They are classically expressed in response to viral and bacterial infection but are also induced by a variety of non-infectious inflammatory stimuli, including tumors. The varied responses elicited by the different types of interferons can either have protective or deleterious effects on local tissues. With these two opposing effects, interferons set the balance between tissue homeostasis and the forms of tissue deterioration that characterize many disease states^[1–;3].^ The local concentration of interferons in the tissue microenvironment and the timing of interferon induction are crucial determinants of tissue integrity.

Interferons are classified into three categories based on their receptor binding affinity. Type I interferons, the first to be discovered and the broadest class, encompass more than 20 subtypes of IFNα, as well as IFNβ, IFNε, IFNκ and IFNω. These molecules commonly engage the heterodimeric IFNα receptor (hereafter referred to as IFNAR). While most cells are capable of producing IFNα/β to some extent, the largest proportion of these cytokines is produced by specialized immune cells.

The Type II class is represented by a single protein, IFNγ, which signals through the IFNγ receptor complex IFNGR1/IFN-GR2 (hereafter referred to as IFNGR). IFNγ is produced primarily by immune cells, but its receptor is virtually ubiquitous. Robustly induced after infection, IFNγ exerts effects that are both diverse and widespread, and thus plays a central role in both innate and adaptive immunity.

The most recently discovered class of interferon, and about which the least is known, is type III. The type III class encompasses four distinct IFNλ proteins that are expressed by a broad variety of cell types. These proteins share structural elements with the IL-10 family of cytokines, and engage the dedicated IFNλ receptor IFNLR. In contrast to the widely expressed receptors of type I and type III interferons, IFNLR is primary expressed in epithelial tissues that serve as a protective barrier against pathogens in the external environment. This unique tissue distribution makes the IFNλ signaling pathways particularly relevant to the etiology of common carcinomas, such as CRC.

Induced in the context of infection, interferons induce cell-intrinsic antimicrobial states in both infected and neighboring cells and thereby limit the spread of pathogens. Interferons continue to play an evolving role during the course of infection as they temporally control innate immune responses, promoting antigen presentation and natural killer cell functions while restraining pro-inflammatory pathways and cytokine production. Interferons also promote the development of high-affinity antigen-specific T and B cell responses and immunological memory, and thus activate the adaptive immune system^[1]^.

Inflammation is a critical component of the microenvironment of most common tumors, including CRC. With their fundamentally dual nature, interferons can have both pro- and anti-inflammatory effects during tumorigenesis. Pro-inflammatory responses are important for initiating the immune response against abnormal cells, while anti-inflammatory responses appear to be essential for resolution of the inflammatory state and subsequent tissue repair.

Endogenous interferons have been associated with significant antitumor activity^[3,4].^ Cytotoxic effects of interferons can be direct, as in the induction of cancer cell apoptosis, or indirect, via the stimulation of immune cells that affect cancer cell clearance. Interferons can, in certain contexts, also drive the growth of tumors by contributing to the proliferative microenvironment associated with chronic inflammation, and by promoting expression of immunosuppressive molecules such as the immune checkpoint protein programmed death ligand 1 (PD-L1)^[4]^.

It is important to note that the functions of interferons in the context of chronic infection and cancer are essentially the same. This commonality is particularly apparent in the pathogenesis of virus-associated tumors, such as human papillomavirus-associated cancers that arise in the uterine cervix, the anogenital epithelia and the head and neck.

**Fig. 1.**
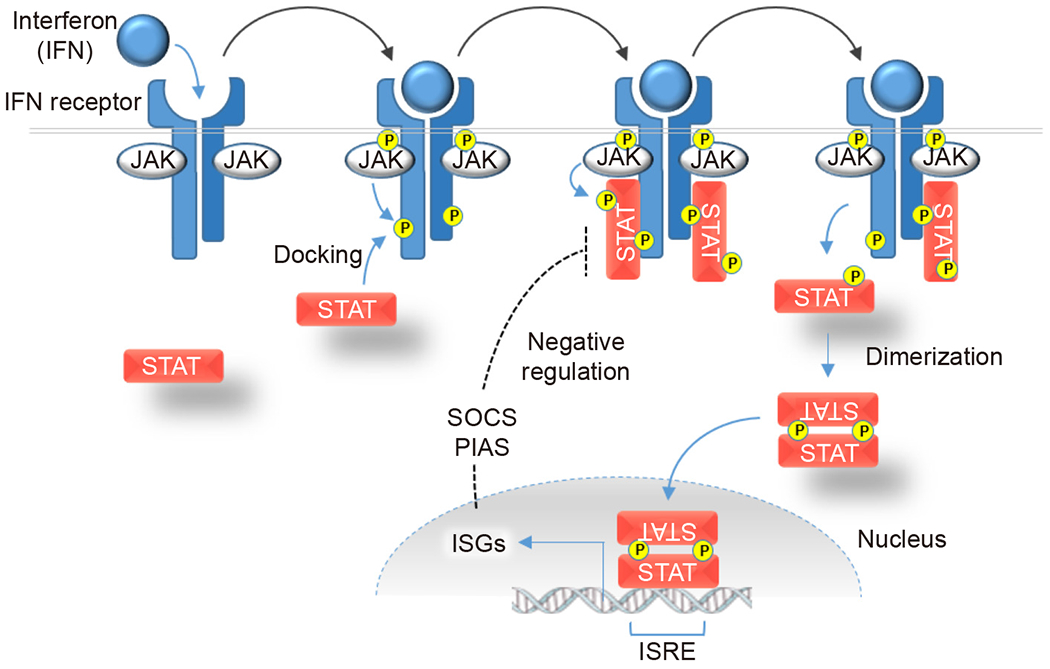
Activation of JAK/STAT signaling pathways. The association of extracellular interferons with their corresponding heterodimeric receptors on the cell surface results in receptor dimerization and JAK activation. Activated JAK proteins phosphorylate tyrosine residues (yellow circles) on the receptor, thereby creating docking sites for monomeric STAT proteins. After docking, STATs are then tyrosine-phosphorylated as well which leads to their dissociation from the receptor to form homodimers or heterodimers composed of different STAT subunits. STAT dimers enter the nucleus and specifically bind to interferon-stimulated response elements (ISREs) in the promoters of downstream ISGs, thus increasing their transcription. Several ISG products, such as the suppressor of cytokine signaling (SOCS) proteins and protein inhibitor of activated STAT (PIAS) proteins inhibit STAT docking and phosphorylation, and thereby form a regulatory feedback loop.

Interferons bind to their respective receptors on the cell surface (Fig. 1). The binding of an interferon to its cognate receptor results in conformational changes in the receptor and the activation of Janus kinases (JAKs). Activated JAKs in turn recruit Signal Transducers and Activators of Transcription (STAT) molecules via phosphorylation.

While signals are initiated by three distinct ligand-receptor complexes, the downstream JAK-STAT signaling pathways are highly interconnected^[5].^ There are four mammalian JAK proteins and seven known members of the mammalian STAT family. Type I and type II interferon/IFNR complexes preferentially interact with several distinct JAK-STAT complexes, some of which are common to both pathways. Our knowledge of the pathways downstream of type III interferons remains incomplete, but it appears that IFNλ signals have very similar downstream effects as the signals generated by interferons in the type I class.

Following their activation by JAKs, the STATs dimerize and translocate to the nucleus, where they directly induce transcription of a large transcriptome collectively known as interferon-stimulated genes (ISGs, see Fig. 1). There is considerable overlap in the downstream targets of the different interferons. Some genes are commonly activated in response to all interferons; others are primarily activated by a single class. The relative abundance of receptors varies considerably among cell types, which not only permits differing levels of sensitivity but also causes variation in the distinct sets of ISGs that are reduced in response to a given interferon.

Hundreds of ISGs are encoded in the mammalian genome. Their protein products ultimately execute a remarkable number and variety of antiviral and immunomodulatory functions^[2,6]^. As the first line of defense against pathogen infection, ISGs collectively antagonize virtually every stage of virus propagation. ISG-encoded proteins serve central roles in diverse processes that include pathogen sensing, inhibition of viral entry, degradation of pathogen-associated RNAs, modulation of cellular metabolism, regulation of the cell cycle and apoptosis. Importantly, some ISGs also function to downregulate JAK-STAT signaling, thereby forming a crucial negative feedback loop that allows the intensity of the immune responses to be highly dynamic (Fig. 1).

## Interferon signaling, inflammation and tumorigenesis

Given their remarkable range of biochemical functions and their central roles in immune regulation, it is perhaps not surprising that interferons and ISGs are intimately involved in tumorigenesis. Sustained interferon pathway induction is strongly associated with inflammation, a cardinal component of the immunosuppressive tumor microenvironment. Cytotoxic (CD8 + ) T cells and Type 1 T helper (Th1) cells, which together form the lynch-pin of immune surveillance, are downregulated in this unique niche^[7]^.

The induction of type I interferon in infected tissues initially promotes beneficial T cell responses, but prolonged exposure to these signals leads to T cell hyperactivation followed by exhaustion^[8]^. The pro-inflammatory effects of aberrant type I interferon signaling are underscored by the close association between this class of cytokines and a broad spectrum of inflammatory illnesses, including systemic lupus erythematosus, rheumatoid arthritis, systemic sclerosis and juvenile dermatomyositis^[9]^. Among the inflammatory ISGs induced in responses to IFNα, are genes that encode the chemokines CXCL9, CXCL10, and CXCL11^[10]^. Exported to the tumor microenvironment, these chemokines recruit T cells, natural killer (NK) cells, and inflammatory monocytes.

The role of IFNγ in tumorigenesis is more broad and perhaps more complex; concomitant pro- and anti-tumor functions have been described^[11]^. Nonetheless, it is well established that prolonged exposure to IFNγ suppresses effector T cell function via several mechanisms, including the downregulation of major histocompatibility complexes and upregulation of the checkpoint protein PD-L1. IFNγ is a potent activator of indoleamine 2,3-dioxygenase, an enzyme required for the generation of kynurenine and related immunomodulatory compounds that pass into the microenvironment and suppress T cell activity^[12]^. Mazet et al.^[13]^ have recently demonstrated that chronic exposure to IFNγ specifically suppresses the proliferation of stem-like T cells, which ultimately leads to a loss of clonal diversity.

The relationship between the type III interferons and inflammation, and its potential relevance to cancer, remains to be fully elucidated. Numerous studies suggest that IFNλ is a relatively weak stimulus for inflammation. While the type I and type III interferons activate very similar transcriptional programs, the ISG transcriptome induced by IFNλ notably lacks CXCL9, CXCL10, and CXCL11. Interestingly, Forero et al.^[10]^ demonstrated that the differential expression of these genes downstream of type I and type III interferons could be attributed to a distinct inflammatory response generated by the transcription factor IRF1, which in their model system was only induced in response to type I interferon. Overall, the response to type III interferon is less potent, compared to the high levels of ISGs that are induced in response to type I interferon, and exhibits slower kinetics.

## Temporal modulation of inflammation in the colorectal epithelia

**Fig. 2.**
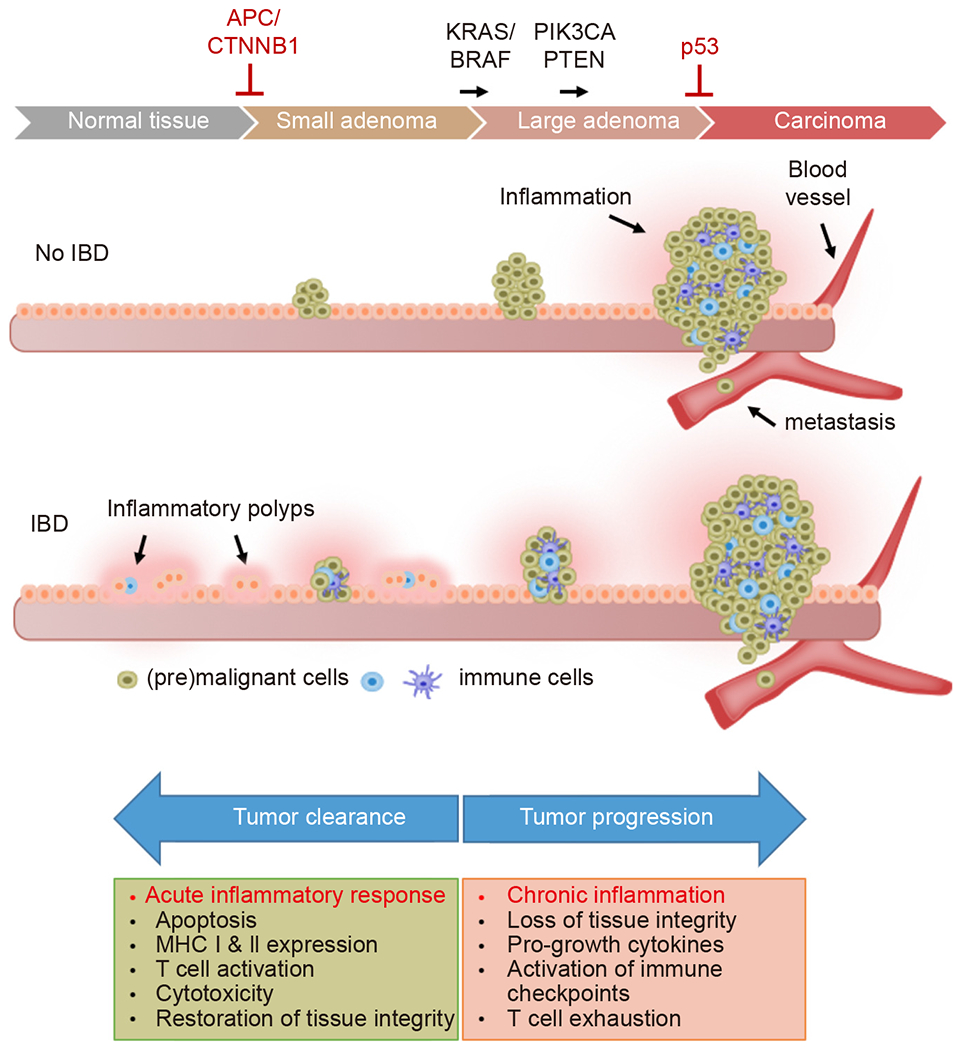
Inflammation during colorectal tumorigenesis. Tumors in the colorectal epithelia arise in stages that are each associated with recurrent genetic alterations, as indicated. The mutational inactivation of the *adenomatous polyposis coli* (*APC*) gene, or mutational activation of the gene that encodes the APC associated protein β-catenin *(CTNNB1)*, initiates the initial stages of tumor development resulting in the formation of small adenomas. The growth and molecular evolution of adenomas is subsequently facilitated by the activation of RAS signaling, either through mutation of *KRAS* or *BRAF*, and constitutive activation of the regulatory pathway modulated by the opposing functions of *phosphatase and tensin homolog* (*PTEN)* and *PIK3CA*. Loss of the tumor suppressor p53 in large adenomas causes them to become invasive carcinomas. The tumors that arise in otherwise healthy tissues typically become inflamed (red glow) at this later invasive stage. In the setting of IBD, chronic inflammation distorts the normal cell architecture and is a component of all lesions that arise. Ulcerative colitis causes increased rates of tumorigenesis in the colon epithelia and is also associated with the formation of distinct lesions called inflammatory polyps (also known as ‘pseudopolyps’). These lesions are also composed of epithelial and stromal tissues but have low malignant potential. As illustrated, interferons can alternatively drive tumor growth or tumor clearance, depending on the quality, intensity and duration of downstream signals.

The etiologic relationship between inflammatory bowel disease (IBD) and CRC provides a paradigm for understanding the role of chronic inflammation in cancer. Ulcerative colitis, the form of IBD that is restricted to the colon and rectum, is characterized by a relapsing and remitting course, marked by inflammatory cell infiltration and immune-mediated tissue damage (Fig. 2). Patients with ulcerative colitis are at an elevated risk of developing CRC; the relative risk increases with the duration of inflammation^[14,15]^. While the incidence of IBD has been increasing in recent years, the cancer risk associated with ulcerative colitis has been on the decline, a positive development that has been attributed to improved clinical management of chronic inflammation and improved endoscopic surveillance.

It has become increasingly clear that gut health is largely determined by complex interactions between the mucosal immune system and commensal bacteria. In this precarious immunologic environment, interferons act as important cross-regulators that maintain an optimal level of antimicrobial activity. Interferons can exert pro- or anti-inflammatory effects to maintain an appropriate balance, depending on the composition of the microbiome and the status of the epithelial barrier. Recent studies have provided new insight into the role of type I interferons in maintaining tolerance to the microbiota that inhabit the gut^[16]^.

The development of IBD involves the breakdown of two anatomical barriers, the gut epithelium and the more recently recognized barrier presented by the vascular endothelium^[17]^. Chronic IFNγ signaling, typically induced in the setting of bacterial infection, can compromise both of these barriers and is therefore an important driver of IBD pathogenesis^[18,19]^. Notably, ulcerative colitis sometimes develops following a transient bacterial infection, suggesting that the etiology of this disease involves a failure of IFNγ downregulation after pathogen elimination. Indeed, distinctive patterns of ISG expression are prospective biomarkers for quantifying the extent of disease in the clinical setting^[20]^.

Groundbreaking studies in mouse models of chemically-induced colitis have affirmed the importance of the vascular endothelial barrier and provided new insights into the molecular mechanisms by which IFNγ signaling can break it down. Treatment of mice with dextran sodium sulfate causes acute intestinal injury followed by gradual recovery and the restoration of homeostasis. Langer *et al.*^[21]^ found that IFNγ caused vascular barrier permeability following chemical injury through disruption of VE-cadherin, the major endothelial adhesion molecule that maintains cellular junctions and controls vasculogenesis^[22]^. This effect was ameliorated in mice harboring an endothelial-specific knockout of the *Ifngr2* receptor. Naschberger *et al.*^[23]^ have recently identified an IFNγ-induced protein secretome, produced by human endothelial cells in culture, that provides further insight into the molecular mechanisms by which vascular permeabilization can occur.

Type I interferons, until recently more commonly associated with viral rather than bacterial infections, also mediate homeostasis in the tolerogenic environment of the colorectal epithelia. Studies across several mouse models of experimental colitis initially suggested that type I interferons primarily exert anti-inflammatory effects^[24,25]^, findings that were somewhat at odds with observations in human subjects. More recent murine studies have supported a more nuanced model in which type I interferons are anti-inflammatory shortly after injury but pro-inflammatory as damaged tissues recover and regenerate^[25]^.

The distinctive distribution of the type III interferon receptor IFNLR in barrier epithelia, including the lining of the gastrointestinal tract, suggests that IFNλ also plays a role in the homeostasis of the colorectal mucosae. Recent studies in mice have borne out this prediction. Type III interferons, like the type I class, are primarily associated with viral infection, but appear to function in a predominately anti-inflammatory capacity^[25]^. In the mouse, IFNλ can be elicited by enteric viruses as well as dextran sodium sulfate, and has the unique ability to decrease oxidative stress and thereby limit intestinal tissue damage by preventing the degranulation of neutrophils^[26]^. In apparent opposition to type I interferons, IFNλ exerts protective, anti-inflammatory effects during recovery from acute injury in mice. An integrative model in which type I and type III interferons work collaboratively to restore homeostasis is supported by recent studies of double knockout (*Ifnar1*^−/−^*Ifnlr1*^−/−^) mice^[27]^. After chemical injury, these mice exhibit enhanced tissue destruction, with extensive loss of goblet cells and diminished proliferation of epithelial cells. The emerging picture is one in which type I and type III interferons play complementary roles after intestinal injury to ensure regeneration of the intestinal epithelium and timely restoration of the mucosal barrier.

## The impact of interferons during colorectal tumorigenesis

While chronic inflammation creates a proliferative environment that drives tumor progression, acute inflammatory responses against damaged, neoantigen-presenting cells are essential for effective anticancer immune surveillance^[28,29]^. Therefore, the duration of an inflammatory response against a cancer precursor lesion largely determines whether tumor growth will be promoted or suppressed. Acute inflammatory responses stimulate the maturation of dendritic cells (DCs), which enhance antigen presentation and form the key link between innate and adaptive immunity^[30]^. An acute inflammatory response that fails to eliminate a targeted neoplasm (or a targeted pathogen) effectively sets the stage for chronic inflammation. The attendant onset of localized immune suppression allows surviving cell populations to continue to proliferate, increasing the risk that subclones will acquire additional mutations that can drive continued evolution into a cancer.

The diametrically opposing effects of acute and chronic inflammation explain the dual nature of interferons in tumorigenesis. While chronic exposure of colorectal epithelia to IFNγ is a key driver of IBD – and thus a major risk factor for CRC - the robust induction of IFNγ signaling results in the elimination of neoplastic cells and thereby functions as a critical suppressor of tumorigenesis.

In a seminal study published 30 years ago, Dighe *et al.*^[31]^ were the first to demonstrate that IFNγ-insensitive tumor cells exhibited accelerated growth in syngeneic mice. In the years since, studies of mouse models of cancer and human cancer patients have offered a wealth of evidence that the interferon signaling pathways collectively function as an extrinsic suppressor of tumorigenesis^[32]^. The essential role for IFNγ in anticancer immune surveillance has been demonstrated across a variety of preclinical models^[28]^. Transplanted colon tumors were found to grow more rapidly in *Stat2^−/−^* mice, in which infiltrating DCs and CD8 + T cells were clearly deficient^[33]^. The conditional knockout of IFNGR in intestinal epithelial cells was recently shown to markedly enhance spontaneous and colitis-associated tumorigenesis in mice, providing conclusive evidence for the anticancer role of IFNγ in CRC^[34]^.

At least some of these anticancer effects appear to persist through the later stages of tumorigenesis. In established CRCs, an IFNγ-driven ISG expression signature has been found to correlate with the infiltration of Th1 helper cells and cytotoxic T cells as well as increased patient survival^[35]^. Conversely, the loss of IFNGR expression in tumor cells can predict poor prognosis in patients with CRC^[34,36]^.

The impact of IFNγ on CRC risk and patient survival is further supported by molecular epidemiology. Slattery et al.^[37]^ examined the distribution and impact of genetic variants in IFNGR and other components of the pathway in a case control study involving more than 2000 CRC patients. This study revealed that variation in the genes that encode IFNγ, IFNGR and several downstream effectors were statistically associated with cancer risk and survival after diagnosis.

The precise mechanisms by which IFNγ impairs tumor growth are subject to active investigation. Among the ISGs robustly induced by IFNγ in a variety of cell types is GBP1, which encodes the guanylate-binding protein 1. GBP1 restrains cell proliferation in the inflammatory microenvironment, and therefore provides a direct mechanistic link between IFNγ and CRC cell growth^[38]^. GBP1 expression is absent in many CRC cell lines, and its loss in tumors has been associated with acquired resistance to IFNγ, which is often observed in late stage, metastatic disease^[39]^.

Defined roles for the type I interferons in CRC development have emerged relatively recently^[40,41]^. It has long been understood that type I interferons interact with tumor cells directly, inhibiting their growth by promoting both cell cycle arrest and apoptosis. However, the effects of type I interferon signaling on the T cells in the tumor microenvironment appear to be the more predominant factor. Type I interferons enhance the suppressive effects of tumor associated, but not systemic, Tregs in CRC^[42]^. IL-10 production by this Treg population was shown to be dependent on IFNAR1 signaling, suggesting that local type I interferon product drives a tumor-suppressive Treg phenotype^[42]^. Downregulation of the type I interferon receptor chain IFNAR1, a phenomenon observed in mouse models of CRC and in human CRC patients, in effect creates an immune-privileged niche that promotes disease progression^[43]^.

While they remain relatively understudied in this regard, type III interferons also appear to exert effects on the tumor microenvironment that on balance appear to inhibit cancer progression. In most experimental models employed thus far, IFNλ appears to effectively coordinate innate immune responses with minimal collateral inflammation^[43]^. Interestingly, subsets of DCs actively produce IFNλ in the breast tumor microenvironment, and thereby contribute to local Th1 activation. Accordingly, the expression of IFNλ and its cognate receptor have been associated with favorable outcomes in breast cancer cohorts^[44]^. Whether IFNλ plays a similar role in CRC remains to be determined.

An emerging body of data suggests, that IFNλ, like the other interferons, might also have pro-tumor effects in some settings. Endogenous expression of IFNλ has been linked to poor prognosis in cancer patients, with the genes that encode IFNλ2 and IFNλ3 serving as independent prognostic markers^[45].^ Increased immune cell infiltration has been observed in patients with elevated IFNλ expression, but has not been correlated with improved prognosis. It remains unclear whether the observed increase in IFNλ expression drives tumorigenesis to a significant extent, or is merely a consequence of aggressive tumor growth.

Consistent with the dichotomy that has become apparent in the complex relationship between interferons and cancer, several ISGs exhibit both anti-tumor and pro-tumor effects in the colorectal epithelia. The IFITM family, composed of five clustered genes that encode small transmembrane proteins, is a notable class of ISGs in that their encoded proteins are trafficked to the cell surface where they contribute to the tumor microenvironment. IFITM1, IFITM2 and IFITM3 are induced by all types of interferon and are overexpressed in various tumor types, including CRC. Their expression levels are generally predictors of poor prognosis and are positively correlated with CRC metastasis^[46–49]^. IFITM3 is upregulated in ulcerative colitis^[50]^ and in precancerous adenomas^[51]^ and has therefore come into focus as a potential driver of CRC progression^[48]^.

Despite their positive association with cancer, IFITM proteins exhibit activities that on balance would be more consistent with an anticancer role. The pleiotropic functions of the IFITM proteins include translational regulation^[49]^. Genetic knockout of IFITM1 and IFITM3 causes attenuated expression of several ISGs, including the human MHC Class I protein HLAB as well as other ISGs^[52]^. These findings suggest that IFITMs may inhibit tumorigenesis by promoting antigen presentation, a prerequisite for immunosurveillance.

Notably, IFITM3 expression is also regulated independently of interferons, by the tumor suppressor KLF4^[53]^. While the exact role of IFITM3 and the other IFITM family members in tumorigenesis remains the focus of intensive investigation, these findings suggest that CRC progression is not simply related to the intensity of ISG induction, but rather the precise composition of the ISG program, the influence of other regulatory pathways, and ultimately, the net effects of many positive and negative determinants of tumor growth.

## Interferons and cancer therapy

Interferons were the first biologics to be developed for cancer therapy^[3,54]^. Given their pleiotropic effects and central role in modulating immune responses, there was early hope that the type I interferons would effectively be ‘magic bullets’ that could stimulate antitumor immune responses while directly inhibiting cancer cell proliferation and survival^[4]^. As is the case all too often, these early sentiments have proven overly optimistic. Efforts to develop interferons as therapies for CRC and other tumors are ongoing, but experience thus far has shown that the clinical effects of the type I interferons are complex, difficult to predict and too often associated with dose-limiting toxicities.

Gresser et al.^[55]^ demonstrated in 1969 that purified interferon preparations could prolong survival in mice with implanted tumors. In 1986, IFNα2 became the first immunotherapeutic agent to be approved by the FDA, and the second approved drug, after insulin, to be produced by recombinant DNA technology. Initially approved for use in melanoma, IFNα2 was subsequently associated with tumor regression and/or prolonged survival in a diverse hematological and solid malignancies including myeloma, lymphomas, renal cell and bladder carcinomas and Kaposi sarcoma^[54]^.

While IFNα2 and other type I interferons showed promising anti-tumor effects, it soon became clear that systemic administration was associated with severe adverse outcomes, including cytopenias, fatigue, anorexia, hepatotoxicity, flu-like symptoms and severe depression with suicidal ideation^[56,57]^.

Recent efforts to circumvent these side effects have been largely focused on targeted delivery to specific cell populations and microenvironments, thereby avoiding systemic toxicity, and using lower interferon doses in combination with other therapies^[3,58]^. An attractive alternative to administering type I interferons directly is to stimulate the production of endogenous type I interferons with prospective drugs, such as agonists of the cGAS/Stimulator of Interferon Genes (STING) pathway ^[59]^.

Another therapeutic target is IFNAR. In mouse and human CRC cells, the expression of IFNAR is downregulated. Genetic stabilization of IFNAR has been found to improve T cell cytotoxicity, thereby increasing the efficacy of chimeric antigen receptor T (CAR T) cell transfer and immune checkpoint blockade (ICB)^[43]^. These findings suggest that agents that increase IFNAR expression or inhibit its turnover could be useful in combination with existing immunotherapies.

Like the type I interferons, IFNγ has dose-limiting side effects that have largely precluded the widespread adoption of this cytokine as a treatment for solid tumors. While endogenous IFNγ signaling is clearly important for immune surveillance, high-dose administration of exogenous IFNγ strongly stimulates PD-L1 expression in the tumor microenvironment, and hampers therapy with agents that enforce ICB^[41]^. Studies that have explored the use of IFNγ as a surgical adjuvant or a in combination with other biologics for treatment of patients with high-risk CRC have yielded disappointing results^[60,61]^. Preliminary studies have suggested that low dose regimens designed to minimize toxicities can induce antitumor responses at the cellular level, but the clinical efficacy of this approach remains to be established^[62]^.

As suggested by studies of the type I interferon signaling pathways, a safer and more effective therapeutic approach to interferon insensitivity may be to increase the abundance of the interferon receptor rather than the interferon itself. Studies of IFNGR1 turnover have revealed that interfering with palmitoylation, the covalent attachment of fatty acids to cysteine and less frequently to serine and threonine residues of proteins, can restore IFNγ signaling in CRC, and thereby enhance T cell activity and sensitize tumors to immunotherapy^[63]^.These preclinical studies suggest that stabilizing IFNGR1 by inhibiting its palmitoylation may be a viable strategy for overcoming resistance to ICB in CRC. Exploring the effects of a different posttranslational modification, Krug *et al* have recently reported that IFNGR expression is regulated in human CRC cells through N-glycosylation, which targets it for proteasome-dependent degradation and thus decreases protein stability. They determined that downregulation of the enzyme N-acetylglucosaminyltransferase III (MGAT3) was highly correlated with low IFNGR expression and IFNγ resistance in CRC tissues. Pharmacological stimulation of MGAT3 expression with all-trans retinoic acid increased N-glycosylation and IFNGR-dependent signaling^[34]^.

Further downstream in the interferon signaling pathways, the ISGs present a plethora of prospective therapeutic targets. Several ISGs have been directly implicated in the pathogenesis and/or response to therapy in CRC. Particularly intriguing among these is ISG15, a small, ubiquitin-like protein that is covalently attached to a broad spectrum of substrates following interferon pathway stimulation, and which has been implicated as a central player in the host antiviral response^[64]^. In cancer cells, induction of ISG15 disrupts protein turnover by the proteasome, and increases the intracellular abundance of ubiquitinated proteins^[65]^. ISG15 is reportedly highly expressed in CRC and closely associated with poor prognosis. Interestingly, ISG15 was reportedly upregulated following trametinib treatment; in this context, genetic knockdown of ISG15 enhanced the efficacy of this treatment^[66]^.

In contrast, the ISGs CCL5 and CXCL10 appear to play a positive role in the effectiveness of therapy. These proteins are upregulated in response to the endogenous activation of cGAS/STING and type I IFN signaling by damaged DNA. In mismatch repair-deficient CRC, which are particularly immunogenic, the recruitment and activation of systemic CD8 + T cells was found to be highly dependent on CCL5 and CXCL10 induction^[67]^. Notably, this study also found that CCL5 and CXCL10 could be upregulated by common chemotherapies in all CRCs, irrespective of their mismatch repair status, suggesting a general pathway to therapeutic sensitization.

Type III interferons, the newest class, largely restricted to barrier epithelia, demonstrate an exciting ability to coordinate the host antiviral response without inducing collateral hyperinflammation. Clinical trials of IFNλ for respiratory viral infection have not shown the high levels of toxicity and adverse effects elicited by other types of interferons^[68]^. The efficacy and safety of IFNλ-based antiviral therapy has recently been further supported by trials in patients with COVID-19^[69]^, the pathogenesis of which involves downregulation of IFNλ signaling^[70]^. IFNλ has also shown a favorable safety and efficacy profile in the treatment of chronic hepatitis C virus infection^[71]^.

Preclinical studies have thus far supported the application of IFNλ as an anticancer agent^[72]^. In several mouse tumor models, IFNλ has been found to upregulate cell surface MHC Class I expression and to impede tumor growth^[73,74]^. Treatment of CRC cells with IFNλ can trigger apoptosis, suggesting that some of its antitumor effects in the colon may be direct^[75]^.

The distribution of IFNLR in intestinal epithelial cells suggests that IFNλ might be particularly useful for the treatment of chronic inflammation in the gastrointestinal tract, an important risk factor for CRC. Preclinical studies indeed suggest that IFNλ signaling pathways could be a useful target for the treatment of IBD, although some important caveats have been raised^[76]^. Studies in STAT1-deficient mice and in patient-derived colon organoids have shown that IFNλ promotes healing of the colorectal mucosae by activation of STAT1^[77]^. Recently, IL-20 was shown to have a similar healing effect through activation of STAT2, raising the possibility that a combination of both of these cytokines could synergistically increase efficacy^[78]^.

An important consideration regarding the suitability of IFNλ for the treatment of chronic inflammation and, perhaps, CRC is the fact that the precise cellular targets are still being identified. Moreover, the loci that encode IFNλ and IFNLR are polymorphic in humans, and data regarding the impact of genetic variability on this signaling pathway is presently lacking^[79]^.

Murine models, which have greatly contributed to our understanding of the pleiotropic functions of IFNλ and the other interferons, have important limitations in both of these aspects. Mice express two IFNλ genes whereas humans have four. In addition, similar cell types from the two species can exhibit markedly different degrees of IFNλ responsiveness. For example, chimeric mice with human hepatocytes revealed striking species-dependent responses to IFNλ, with mouse hepatocytes being essentially non-responsive^[80]^. The expression of IFNLR in immune cells also seems to also differ significantly between mice and men^[79]^.

## Biomarkers of T cell activation

Secreted by diverse cell types in response to a variety of stimuli, interferons are highly sensitive and specific biomarkers of immune activity. Accordingly, assessments of interferon production can help quantify the effects of immune-stimulating therapeutics.

The most widely used method for enumerating antigen-specific cytokine-producing immune cells is the enzyme-linked immunospot (ELISpot) assay^[81]^. There are many different versions of this assay, but all employ the enzyme-linked immunosorbent assay (ELISA) technique. In this method, either a monoclonal or polyclonal antibody specific for the chosen interferon is precoated onto a microplate. Appropriately stimulated cells (e.g., peripheral blood mononuclear cells) are incubated in the coated wells and the immobilized antibody binds the secreted analyte. The unbound material is washed away and the bound analyte is then specifically detected with a second, biotinylated antibody. In most assays, a chromogenic substrate creates a “spot” of measurable intensity that can be read manually, or via an automated ELISpot reader system.

The enumeration of interferon-producing cells by ELISpot can provide insight into the efficacy of immune-stimulating therapies^[82,83]^, which is particularly valuable during clinical trials. For example, Oh and colleagues^[84]^ used IFNγ as a sensitive measure of T-cell activity in order to assess the immune reactivity of advanced ovarian cancer patients to treatment with an autologous tumor cell vaccine. Their analysis of treated and untreated cohorts revealed that this cancer vaccine increased T cell activation during a phase 2A trial, which correlated with an increase in median survival^[84]^.

The quantification of interferon-secreting cells is now a standard metric in clinical studies of developmental immune therapies for CRC. In their preliminary evaluation of a personalized neoantigen vaccine for treatment of microsatellite-stable CRC, Yu *et al.* recently employed IFN-γ ELISpot to evaluate neoantigen-specific T cell activity^[85]^. In parallel, they employed a variety of clinical assessment tools, including the abundance of neoantigen-associated mutations in circulating tumor DNA. This study demonstrates how the availability of quantitative assays of interferon production and complementary genetic methods for assessing antigenic tumor burden now provide increasingly detailed information regarding tumor dynamics and therapeutic efficacy.

## Conclusions

Interferons are in many aspects a two-edged sword, required for immune surveillance but also an important component of the immunosuppressive tumor microenvironment. This dichotomy is mirrored in clinical trials, which have revealed both striking antitumor effects and equally striking adverse effects that have limited their use. Fortunately, basic studies of interferons and their downstream signaling pathways have begun to reveal alternative strategies for selectively restoring the antitumor effects of interferons that are frequently lost during tumorigenesis. The attributes of the new type III interferons are particularly compelling. Their successful application to the therapy of respiratory infections may blaze a trail for their eventual use in cancers. In the intermediate term, there is considerable optimism that type III interferons will find clinical applications in the treatment of IBD, an important risk factor for CRC, and perhaps other inflammatory disorders. Preclinical studies generally suggest that one or more of the IFNλ isoforms may be suitable for therapy of established CRC, most likely in combination with other agents. Mouse models are extremely powerful, but the translation of these findings to humans may not be straightforward. It is obviously imperative that the design of future clinical trials take these important limitations into account.

## Abbreviations

CAR T cell: Chimeric antigen receptor T cell
CRC: Colorectal cancer
DC: Dendritic cells
ELISA: Enzyme-linked immunosorbent assay
ELISpot assay: Enzyme-linked immunospot assay
IBD: Inflammatory bowel disease
IFNα: Interferon-α
IFNγ: Interferon-γ
IFNλ: Interferon-λ
JAK: Janus kinase
STAT: Signal Transducer and Activators of Transcription
STING: Stimulator of Interferon Genes
